# Vertical Jump Changes Across the Stretch-Shortening-Cycle Continuum Following Complex Training in Adolescent Female Volleyball Players: A Single-Arm Bayesian Analysis

**DOI:** 10.3390/sports14070267

**Published:** 2026-06-26

**Authors:** Kevin A. Rodríguez-Fernández, Marina Trejo-Trejo, H. Antonio Pineda-Espejel, Isaac A. Chávez-Guevara, Arnulfo Ramos-Jiménez

**Affiliations:** 1 Faculty of Sports, Mexicali Campus, Autonomous University of Baja California, Mexicali 21289, Baja California, Mexico; rodriguez.kevin@uabc.edu.mx (K.A.R.-F.); antonio.pineda@uabc.edu.mx (H.A.P.-E.); 2 Faculty of Sports, Ensenada Campus, Autonomous University of Baja California, Ensenada 22890, Baja California, Mexico; isaac.chavez.guevara@uabc.edu.mx; 3 Institute of Biomedical Sciences, Autonomous University of Ciudad Juárez, Ciudad Juárez 32401, Chihuahua, Mexico

**Keywords:** complex training, Bayesian inference, jump performance, adolescent female athletes, volleyball, stretch-shortening cycle, post-activation performance enhancement

## Abstract

Background: Vertical jump performance is a key determinant of volleyball success. Prior evaluations of complex training in youth have relied largely on frequentist statistics, which can be difficult to interpret in the small cohorts typical of youth sport. Objective: Apply Bayesian inference to evaluate vertical jump kinematics across short- and long-stretch-shortening-cycle modalities following a 9-week complex training program in adolescent female volleyball athletes. Methods: Eight athletes completed a periodized training protocol. Jump kinematics were assessed using a high-frequency force platform, and whole-body composition was assessed using bioelectrical impedance analysis. Data were analyzed using Bayesian inference. Results: Bayesian analysis indicated consistent improvements across jump modalities, with Block Volleyball Jump, Countermovement Jump, and Spike Jump heights increasing by 14.4%, 14.4%, and 13.9% (BF_10_ = 29.7, 27.2, and 126.9, respectively), with peak power increasing in parallel for all three jumps (BF_10_ = 22.8, 23.1, and 37.6). Whole-body Bioelectrical Impedance Analysis (BIA) detected no meaningful change in body mass (BF_10_ = 0.34) or in muscle mass percentage (BF_10_ = 1.56) over the intervention period. Conclusions: Complex training was associated with increases in jump height across modalities representing different points on the stretch-shortening-cycle continuum. Bayesian inference provided a complementary probabilistic basis for evaluating training-related change in a small cohort. No accompanying change in whole-body composition was detected by BIA; because muscle architecture was not assessed and the design was single-arm without a control group, the mechanism underlying the jump-height changes cannot be determined.

## 1. Introduction

Volleyball is an explosive, intermittent sport in which athletes perform 100–300 maximal or near-maximal vertical jumps per match, depending on positional role and rally duration [1]. The capacity to generate vertical acceleration and convert horizontal approach velocity into vertical power underpins serving, setting, spiking, and blocking, making vertical jump performance a cornerstone of volleyball strength and conditioning [1,2,3]. The jump is shaped by the stretch-shortening cycle (SSC), which consists of an eccentric action, a brief amortization phase storing elastic energy, and an explosive concentric release facilitated by the stretch reflex [2]. These court actions can be placed along a stretch-shortening-cycle continuum defined by ground-contact time. At the short-SSC end, the Block Volleyball Jump (BVJ) uses a brief ground contact (<250 ms) in which performance depends mainly on tendinous stiffness and rate of force development (RFD). At the long-SSC end, the Countermovement Jump (CMJ) and Spike Jump (SPJ) use a longer ground contact (>300 ms) with deeper joint displacement and, for the SPJ, horizontal approach velocity [1,2].

To enhance SSC efficiency, strength and conditioning programs widely use complex training (CT), the timed intra-session pairing of heavy resistance training with biomechanically homologous plyometric training [3,4]. Its rationale is Post-Activation Performance Enhancement (PAPE): high-force voluntary contractions transiently increase central drive, lower the recruitment threshold of fast-twitch (Type IIa/IIx) motor units, and phosphorylate myosin regulatory light chains, increasing myofilament calcium sensitivity and cross-bridge cycling [5,6]. This potentiated state enhances RFD and peak power and may yield greater chronic adaptation than resistance or plyometric training alone [7,8], and meta-analyses confirm that combined training improves jumping, sprinting, and change-of-direction performance in youth athletes [8,9]. However, prior analyses disagree on whether these adaptations transfer uniformly across short- and long-SSC modalities, motivating further investigation in adolescent female volleyball players.

Analyzing data from small athletic cohorts poses a recognized statistical challenge. In the small samples typical of elite sport (often n < 15), frequentist null-hypothesis significance testing with paired *t*-tests or repeated-measures ANOVA is underpowered, which increases the risk of Type II errors and Type M (magnitude) errors—the over- or underestimation of the true effect size that is common in small samples—and can undermine replicability [10]. Bayesian inference offers a complementary approach for such samples. By combining a prior distribution with the observed data through Markov Chain Monte Carlo sampling, it yields direct probabilistic statements about an effect, including the posterior probability that a change exceeds a Smallest Worthwhile Change (SWC) and a Bayes Factor (BF_10_) that expresses the relative evidence for competing hypotheses [11]. We adopt this framework as an analytical choice suited to small samples, not as a claim that it is generally superior to frequentist methods.

Despite extensive evidence that complex training improves jump performance, an important gap persists: whether its benefits transfer uniformly across the short- and long-SSC continuum or are confined to specific movement vectors. This study addresses that gap by applying Bayesian inference to vertical jump kinematics in adolescent female volleyball players, a population and analytical approach seldom combined. We therefore ask whether a 9-week periodized complex training program is associated with improvements across vertical jump modalities that represent different points on the short- to long-SSC continuum in this population.

We hypothesized that the analysis would show evidence of improvements across vertical jump modalities, with high posterior probabilities of exceeding the SWC for both the short-SSC block jump and the long-SSC countermovement and spike jumps, in contrast to prior reports of more task-specific responses in youth.

## 2. Materials and Methods

### 2.1. Experimental Design and Cohort Selection

This investigation used a single-arm, pre–post quasi-experimental design to examine changes in jump and body-composition outcomes over a complex training program in adolescent athletes; because there was no control group, these changes cannot be attributed to the program alone. The initial cohort comprised a girls’ high school volleyball team. After applying the adherence criteria below, eight female volleyball players (mean age 16.3 ± 0.1 years; competitive experience 2.9 ± 0.7 years) met all criteria and were included in the final analysis. No control or comparison group was studied; all analyses are therefore within-group, pre- versus post-intervention comparisons.

Inclusion criteria required a minimum of two years of continuous competitive volleyball experience and the medically verified absence of severe musculoskeletal, neuro-articular, or systemic injury in the preceding six months. Exclusion criteria removed any participant whose training adherence or session attendance fell below 85% of the prescribed sessions to maintain intervention fidelity.

The Ethics Committee and Internal Review Board of the Faculty of Sports at the Autonomous University of Baja California approved the study protocol (Protocol Code: UABC-149/2/C/64/24). The protocol was conducted in accordance with the ethical guidelines of the World Medical Association’s Declaration of Helsinki. Written informed consent was obtained from the legal guardians of all minor participants before baseline testing, and documented verbal assent was obtained from the athletes themselves.

Although a final sample of n = 8 is typically considered underpowered for classical null-hypothesis significance testing, Bayesian inference does not rely on asymptotic assumptions or large-sample sampling distributions [11]. The Bayesian approach accounts for the uncertainty associated with small samples by producing wider, more conservative credible intervals. Nevertheless, n = 8 remains a small sample: credible intervals are correspondingly wide, posterior estimates are sensitive to the choice of prior, and the present findings should be regarded as preliminary and in need of replication in larger, controlled samples [11].

### 2.2. Study Design

Participants completed a periodized 9-week Complex Training (CT) program integrated into the team’s general physical preparation phase (a 3-week anatomical adaptation mesocycle, a 3-week structural overload mesocycle, and a 3-week maximal-intensity mesocycle). Training was conducted twice weekly (Tuesdays and Thursdays) for 85 min per session. A minimum 48 h recovery interval was enforced between consecutive sessions to allow for central nervous system recovery, metabolic clearance, glycogen replenishment, and dissipation of peripheral neuromuscular fatigue [4].

The protocol used contrast loading, combining heavy resistance Weight Training (WT) with biomechanically similar Plyometric Training (PT) in sequential order. Each session included five strength exercises followed by five plyometric exercises. To elicit the intended PAPE effect, all WT sets were completed before the corresponding PT sets, separated by a 5 min passive rest interval. This interval allows for the dissipation of acute localized metabolic fatigue (e.g., clearance of inorganic phosphate and hydrogen ions) while maintaining central neural excitation, motor pool excitability, and elevated myosin regulatory light chain phosphorylation [5].

WT relative intensity (% 1RM) progressed linearly across three mesocycles: Weeks 1–3 at 50–60% of 1-Repetition Maximum (1RM) for connective tissue adaptation; Weeks 4–6 at 60–70% 1RM for initial structural overload; and Weeks 7–9 at 70–75% 1RM to maximize neural drive. Core WT movements targeted the prime movers of the vertical jump kinetic chain, including the barbell half-back squat, machine leg press, dumbbell static lunges, and prone hamstring curls.

The PT component progressed from low-intensity, high-ground-contact exercises (e.g., ankle hops, jump-and-reach drills, and submaximal horizontal multi-jumps) in the early weeks to high-intensity, SSC-dominant tasks in the final mesocycle. These tasks included box drops from 45 cm, depth jumps, and sport-specific spike-jump approaches designed to load the series elastic components of the musculotendinous unit and engage the stretch reflex [7].

### 2.3. Body-Composition Assessment

Body-composition evaluations were conducted at baseline (Week 0) and immediately post-intervention (Week 10) under standardized laboratory conditions. All participants reported to the laboratory after a 12 h overnight fast and had abstained from vigorous physical activity, caffeine, and diuretics for at least 24 h before assessment to standardize fluid status [12].

Bioelectrical Impedance Analysis (BIA): Whole-body mass, fat mass, and skeletal muscle mass (SMM) were estimated non-invasively using a multi-frequency, eight-point tactile electrode BIA device (InBody 720, Biospace Co., Seoul, Republic of Korea). BIA estimates total body water from the impedance relationship (height^2^/R) across multiple frequencies and infers total muscle mass [12]. While practical and efficient for epidemiological screening, its validity for detecting small localized changes in muscle in lean youth athletes is contested, as minor fluid shifts can affect the estimation algorithms [12,13]. Standing height, used in the impedance estimation, was recorded with a calibrated Seca 213 stadiometer (Hamburg, Germany).

### 2.4. Biomechanical Evaluation of Vertical Jumps

To assess SSC kinematics, stretch-reflex potentiation, and vertical force production, the athletes performed three vertical jump modalities relevant to volleyball: the CMJ, the BVJ, and the SPJ [1]. Jumps were captured on an AMTI ACG-0 ACCUGATE force platform (Advanced Mechanical Technology Inc., Watertown, MA, USA). Ground reaction force data were sampled at 1000 Hz and processed via AMTI NetForce software 1.0.

Following a standardized dynamic warm-up and a force-platform familiarization phase to control for learning effects, players performed three maximal repetitions of each jump with a 30 s inter-repetition rest and a 2 min inter-modality rest to limit neuromuscular fatigue.

- Countermovement Jump (CMJ): Participants began in an upright stance, performed an eccentric countermovement to a self-selected depth, and then jumped vertically with maximal effort using a double-arm swing. The CMJ is a standard assessment of long-SSC neuromuscular capability [1].
- Block Volleyball Jump (BVJ): Mimicking defensive net play, participants started in a pre-tensioned semi-squat with their hands at chest height and jumped vertically without an arm swing, isolating lower-limb short-SSC propulsion and vertical rate of force development (RFD) [1].
- Spike Jump (SPJ): Emulating offensive attack mechanics, athletes used a 3- to 4-step approach, translating horizontal velocity into vertical displacement through a long SSC amortization phase and a double-arm upward swing [1].

Total absolute flight time (t) and the specific ground reaction forces were directly utilized to mathematically calculate the maximal vertical jump absolute height (h) using the rigorously standardized ballistic flight equation:

h = (g · t^2^)/8

where g is the acceleration due to gravity (9.81 m/s^2^). This equation assumes that the centre of mass is at the same vertical position at take-off and landing. This assumption is satisfied for the standing CMJ and BVJ but is only approximately met for the SPJ, whose multi-step approach can alter body configuration between take-off and landing. If the centre of mass is higher at landing than at take-off, the flight-time method systematically underestimates true jump height; SPJ values should therefore be read as flight-time-derived estimates and compared chiefly within, rather than across, jump modalities. Jump height was the primary outcome for all three jumps. Because flight time and jump height are related by a fixed expression (h = g·t^2^/8), they carry the same information, so flight time is not reported as a separate outcome. Peak power output (W), derived from the force–time and velocity–time records, was analyzed as a secondary outcome for each jump using the same Bayesian procedure.

### 2.5. Advanced Bayesian Statistical Analysis

All biomechanical and body-composition data were analyzed within a Bayesian framework. Pre- and post-intervention measures were compared with the Bayesian paired-samples (JZS) *t*-test, in which a Jeffreys–Zellner–Siow prior is placed on the standardized effect size; this is the default test implemented in the R BayesFactor package (R Mac version 4.5.2) and was chosen for transparency and reproducibility [11]. For each outcome, the Bayes Factor was computed in both directions: BF_10_ (evidence for a change) and its reciprocal BF_01_ (evidence for no change), so that support for the null could be quantified rather than merely inferred from a non-significant result. Posterior distributions for the standardized effect size (δ) and the raw mean change were estimated by Markov Chain Monte Carlo sampling using four independent chains of 5000 draws each (20,000 posterior samples in total) after 2000 warm-up iterations per chain. This sampling was expanded from the smaller run used in the original submission to obtain more stable posterior summaries and formal convergence diagnostics.

A Cauchy prior with scale r = 0.707 (the BayesFactor package default) was placed on the standardized effect size; this prior treats small-to-moderate effects as most plausible while still allowing larger effects [11]. Bayes Factors were interpreted using the conventional bands described by Jeffreys [14]: 1–3 anecdotal, 3–10 moderate, 10–30 strong, 30–100 very strong, and greater than 100 decisive evidence. To test whether the conclusions depended on this prior, every analysis was repeated across a range of prior scales (r = 0.5, 0.707, 1.0, and 1.414); the resulting Bayes Factors are reported in Supplementary Table S1. Across all priors, the evidence for a change in each jump height remained at least strong (BF_10_ > 10), and the body-composition outcomes remained anecdotal (BF_10_ close to 1); as expected, the precise evidence band for the jumps shifted upward (from strong to decisive) as the prior scale widened, but the qualitative conclusions were unchanged.

To assess practical relevance, the SWC was defined a priori as 0.2 × the baseline between-subjects standard deviation of each measure [11], and the posterior probability that the mean change exceeded the SWC was computed for every outcome (Table 2). For the jump-height and peak-power outcomes, the posterior probability that the effect was in the hypothesized direction, *p*(δ > 0), is also reported. Convergence was assessed with the Gelman–Rubin statistic (R-hat) and the effective sample size (ESS); all parameters reached R-hat = 1.00 with ESS > 4000. Analyses were performed in R (BayesFactor package) and independently reproduced in Python (PyMC version 3.14.6); the dataset and analysis scripts are available from the corresponding author on request.

## 3. Results

### Bayesian Inference of Biomechanical Adaptations

The Bayesian analysis of the 9-week periodized complex training protocol was associated with consistent increases in jump height across all three vertical jump modalities. Table 1 summarizes the descriptive statistics, Bayes Factors (BF_10_), and posterior effect sizes (δ) with their 95% credible intervals (CrI), and Table 2 reports, for every outcome, the posterior probability that the mean change exceeded the SWC.

The jump-height data show increases at both ends of the SSC continuum, rather than at a single point on it. For the short-SSC Block Volleyball Jump (BVJ), height rose from 26.4 ± 3.9 cm to 30.1 ± 3.8 cm, a 14.4% increase, with strong evidence for a change (BF_10_ = 29.7). The posterior median standardized effect was large (δ = 1.35, 95% CrI 0.31 to 2.55) and the probability that the effect was positive was 0.997. The posterior mean increase was 3.43 cm (95% CrI 1.23 to 5.35), and the probability that this change exceeded the SWC of 0.79 cm was 0.99 (Table 2). The wide credible interval reflects the small sample: although the direction of the effect is well supported, its precise magnitude remains uncertain. BVJ peak power increased in parallel, from 634 ± 87 W to 683 ± 88 W (+7.7%), with strong evidence for a change (BF_10_ = 22.8; δ = 1.27, 95% CrI 0.27 to 2.41).

The long-SSC jumps changed by a similar relative amount. The Countermovement Jump (CMJ) increased from 26.9 ± 4.1 cm to 30.8 ± 4.7 cm (+14.4%), with strong evidence for a change (BF_10_ = 27.2; δ = 1.33, 95% CrI 0.30 to 2.52; *p*(δ > 0) = 0.997). The Spike Jump (SPJ) increased from 30.7 ± 4.5 cm to 34.9 ± 4.8 cm (+13.9%), with decisive evidence (BF_10_ = 126.9; δ = 1.84, 95% CrI 0.56 to 3.43; *p*(δ > 0) = 0.999). For both jumps, the posterior probability that the mean change exceeded the SWC was at least 0.99 (Table 2). Because SPJ height is estimated from flight time, and a multi-step approach may raise the centre of mass at landing, the absolute SPJ values may be underestimated and are best compared within, rather than across, jump types. Peak power increased in parallel for both jumps (CMJ: 645 ± 93 W to 693 ± 90 W, +7.5%, BF_10_ = 23.1; SPJ: 690 ± 96 W to 737 ± 87 W, +6.9%, BF_10_ = 37.6), in the same direction as the height changes (Table 1).

Body composition, assessed by multi-frequency bioelectrical impedance analysis (BIA), changed little over the intervention. Body mass was essentially unchanged (56.7 ± 9.4 kg to 56.8 ± 9.6 kg; BF_10_ = 0.34, i.e., BF_01_ ≈ 3.0, favouring no change). Muscle mass percentage shifted only marginally (38.4 ± 3.5% to 38.0 ± 3.7%; BF_10_ = 1.56) and fat mass percentage likewise (29.4 ± 6.7% to 30.0 ± 6.9%; BF_10_ = 1.07); for both, the evidence was anecdotal and the posterior probability of exceeding the SWC was 0.03 or lower (Table 2). These BIA results indicate that whole-body mass and composition were stable, but they should not be interpreted as evidence regarding muscle- or tendon-level changes that may accompany early strength and power adaptation, which BIA cannot resolve [12,13].

## 4. Discussion

This Bayesian analysis indicates that a 9-week periodized complex training program integrating heavy resistance training with biomechanically homologous plyometric exercises was associated with improvements in vertical jump performance among adolescent female volleyball players. The Bayesian analysis addresses a common assumption in the youth training literature: that complex training transfers only to highly specific movement vectors, rather than across the stretch-shortening cycle spectrum. The present results are instead consistent with improvements across all three jump modalities, spanning the short- and long-SSC ends of the continuum, although a single-arm design cannot establish that the training caused these changes.

### 4.1. Transferability of Neuromuscular Improvements Across the SSC Continuum

A key feature of the present dataset is the consistency of the pre-to-post change across jump types. Rather than a disproportionate response favouring the short-SSC Block Volleyball Jump over the Countermovement Jump, the Bayesian analysis (Table 1) showed a comparable 14.4% increase in both BVJ and CMJ height, along with a 13.9% increase in the Spike Jump.

One interpretation of this consistency is that complex training raised the baseline capacity of the motor system rather than refining a single movement pattern, although the present design cannot establish mechanism. Heavy resistance loading (half-squats and leg presses at 70–75% 1RM) is thought to impose mechanical tension and central nervous system drive that may lower the recruitment threshold for high-threshold (Type II) motor units. Pairing this state with both short-SSC and long-SSC plyometric actions is hypothesized to improve force generation and RFD across movement patterns [5,7]. Because the BVJ generates force largely through rapid amortization and tendon stiffness [2], whereas the CMJ and SPJ involve deeper joint flexion and longer muscle–tendon excursion [1], a comparable improvement at both ends of the SSC continuum is consistent with a broad upward shift in force–velocity capacity rather than a change confined to one movement type. These mechanisms are hypothetical: neural drive, motor-unit behaviour, tendon stiffness, and the force–velocity relationship were not measured in this study and cannot be inferred from the jump height data alone.

### 4.2. Advantages of Bayesian Inference in Small Elite-Cohort Analyses

The Bayesian analysis used here offers one way to summarize change in a small sample and is presented as a complement to, not a replacement for, conventional methods [11]. In small athletic cohorts such as the present one (n = 8), frequentist estimates can be unstable and sensitive to sampling variation [10,11]. A *p*-value describes the probability of data at least as extreme as those observed under the null hypothesis, which is not the same as the probability that an effect is present; Bayesian summaries instead express the relative evidence for competing hypotheses and the probability that a change exceeds a predefined threshold [11]. These are general considerations about the two frameworks rather than evidence that one is uniformly preferable.

Using Bayesian paired *t*-tests with a Cauchy prior (r = 0.707), we estimated the evidence for a pre-to-post change in each outcome. For the spike jump, BF_10_ = 126.9 indicates that the observed data are about 127 times more likely under a model that includes a change than under a model with no change [11]. This is a statement about evidence for a change over the study period; because there was no control group, it does not by itself establish that the training caused the change.

The 95% credible interval (CrI) gives a range within which the parameter is likely to lie, conditional on the model and prior. In this study, the credible intervals were wide (Table 1 and Table 2), reflecting the small sample; they should be read as expressing substantial remaining uncertainty about the size of each effect, even where the posterior probability of exceeding the SWC was high. Reporting these intervals alongside the Bayes Factors makes that uncertainty explicit.

### 4.3. Comparison with Previous Studies

The direction and breadth of these changes are broadly consistent with previous complex- and combined-training studies in young athletes, although direct comparison is limited by differences in design, sex, sport, and maturity. In elite male volleyball players, Berriel et al. reported improved jump performance after complex training [5], and in pubertal volleyball players Fathi et al. found that combined strength and plyometric training improved several jump types [3]. Santos and Janeira observed comparable gains in adolescent male basketball players after complex training [4], and a meta-analysis by Ramírez-Campillo et al. reported that plyometric training improves jump height in volleyball players across age groups [7]. The present within-group changes fall in a similar range, but the absence of a control group and the small sample mean that this study should be read only as consistent with, rather than confirmatory of, this body of work.

A distinction should also be drawn between acute and chronic effects. Post-activation performance enhancement (PAPE) refers to a short-lived rise in performance in the minutes after a conditioning activity [6] and was not tested here: no acute potentiation protocol and no repeated within-session measurements were performed. The changes reported in this study were measured across a 9-week program and therefore reflect chronic training adaptation rather than acute potentiation. The complex-training structure pairs heavy and explosive work in the same session and is informed by the PAPE literature [5,6], but the present design does not allow for any acute potentiation effect to be quantified.

### 4.4. Limitations

Although the Bayesian framework mitigates some of the statistical fragility associated with small athletic cohorts, several limitations remain. First, the absence of a matched non-exercising control group precludes full isolation of the training effect from natural maturation (e.g., adolescent growth) and from the concurrent stimulus of sport-specific practice conducted outside the laboratory. Second, jump heights were derived from flight time, which assumes equal take-off and landing centre-of-mass positions and, therefore, yields only approximate values for the approach-based Spike Jump. Third, the single-arm, pre–post design and the homogeneous single-team cohort (n = 8) limit the generalizability of the findings to the broader population of adolescent female volleyball players.

Several biological variables were not controlled across the 9-week window. In particular, the menstrual cycle phase, which can influence joint laxity, tendinous and ligamentous stiffness, and force output, was not tracked longitudinally [1,15]. Biological maturation is another uncontrolled confounder over a 9-week window in adolescents. Differences in maturity status (for example, biological maturity offset relative to Peak Height Velocity) and normal growth can themselves change jump performance; because maturity was not assessed and there was no control group, part of the observed change may reflect maturation rather than the training [9].

### 4.5. Future Directions

Future controlled studies should combine Bayesian models with high-resolution diagnostic ultrasound to track regional changes in muscle architecture—including pennation angle, fascicle length, and cross-sectional thickness—that influence contraction velocity and stretch-shortening-cycle efficiency [12]. Incorporating longitudinal N-of-1 Bayesian hierarchical models may also enable practitioners to track individual responsiveness to PAPE protocols over the course of a competitive season; however, adequately powered, controlled trials that include a comparison group and an assessment of biological maturation will be needed before such individualized prescription can be recommended [11].

## 5. Conclusions

In this single-arm study, a 9-week periodized complex training program was associated with increased vertical jump height in adolescent female volleyball players, with comparable changes across jumps at the short- and long-SSC ends of the continuum (Block Volleyball Jump, Countermovement Jump, and Spike Jump). Whole-body bioelectrical impedance detected no accompanying change in body composition. Because BIA cannot resolve muscle or tendon architecture and the study had no control group, the physiological basis of these changes cannot be determined here.

For practitioners working with small squads, Bayesian estimation offers a useful complementary summary of training-related change by expressing results as the probability and credible range of an effect alongside conventional analyses. These single-arm findings should be confirmed in adequately powered, controlled studies that account for biological maturation before firm training recommendations are drawn.

## Figures and Tables

**Table 1:** Bayesian paired analysis of vertical jump height before and after the 9-week complex training program (within-group comparison, n = 8).

Outcome	Pre (Mean ± SD)	Post (Mean ± SD)	Δ%	δ [95% CrI]	*p* (δ > 0)	BF_10_	Evidence
Block Volleyball Jump height (cm)	26.4 ± 3.9	30.1 ± 3.8	+14.4	1.35 [0.31, 2.55]	0.997	29.7	Strong
Block Volleyball Jump peak power (W)	634 ± 87	683 ± 88	+7.7	1.27 [0.27, 2.41]	0.996	22.8	Strong
Countermovement Jump height (cm)	26.9 ± 4.1	30.8 ± 4.7	+14.4	1.33 [0.30, 2.52]	0.997	27.2	Strong
Countermovement Jump peak power (W)	645 ± 93	693 ± 90	+7.5	1.29 [0.29, 2.46]	0.995	23.1	Strong
Spike Jump height (cm)	30.7 ± 4.5	34.9 ± 4.8	+13.9	1.84 [0.56, 3.43]	0.999	126.9	Decisive
Spike Jump peak power (W)	690 ± 96	737 ± 87	+6.9	1.43 [0.38, 2.73]	0.997	37.6	Very strong

Note: values are from a Bayesian paired-samples (JZS) *t*-test with a Cauchy prior (scale r = 0.707). BF_10_ = Bayes Factor favouring a change; δ = posterior median standardized effect size; CrI = 95% credible interval; *p* (δ > 0) = posterior probability that the effect was positive; Δ% = percentage change from pre- to post-training. Evidence categories follow Jeffreys [14]. Jump height (primary) and peak power (secondary) are reported for each jump; flight time is a fixed transform of height and is not reported separately. All models converged (R-hat = 1.00; effective sample size > 4000).

**Table 2:** Posterior probability that the mean pre-to-post change exceeded the smallest worthwhile change (SWC) for each outcome (within-group comparison, n = 8).

Outcome	SWC	Posterior Mean Δ [95% CrI]	*p* (Change Exceeds SWC)
Block Volleyball Jump height (cm)	0.79	3.43 [1.23, 5.35]	0.99
Countermovement Jump height (cm)	0.82	3.51 [1.20, 5.51]	0.99
Spike Jump height (cm)	0.90	4.00 [1.96, 5.65]	~1.00
Block Volleyball Jump peak power (W)	17.5	44.0 [13.9, 70.6]	0.96
Countermovement Jump peak power (W)	18.6	43.4 [14.4, 68.8]	0.96
Spike Jump peak power (W)	19.3	43.5 [17.0, 66.6]	0.97
Body mass (kg)	1.88	0.03 [−1.09, 1.17]	<0.01
Muscle mass (%)	0.69	−0.31 [−0.71, 0.09]	0.03
Fat mass (%)	1.33	0.47 [−0.22, 1.24]	0.02

Note: SWC = 0.2 × the baseline between-subjects standard deviation of each outcome. The posterior mean change and its 95% credible interval are expressed on the original measurement scale (cm for jump height, kg for body mass, % for muscle and fat mass). For the three jump heights, the probability refers to a change in the hypothesized (upward) direction; for body-composition outcomes, it refers to a change in either sign.

## Data Availability

The data presented in this study are available on request from the corresponding authors. The data are not publicly available due to privacy restrictions concerning minor participants.

## Supplementary Materials

The following supporting information can be downloaded at: https://www.mdpi.com/article/10.3390/sports14070267/s1, Table S1: Prior-sensitivity analysis.

## Abbreviations

The following abbreviations are used in this manuscript:

1RM	1-Repetition Maximum
BIA	Bioelectrical impedance analysis
BVJ	Block Volleyball Jump
CMJ	Countermovement Jump
CT	Complex Training
PAPE	Post-Activation Performance Enhancement
PT	Plyometric Training
RFD	Rate of Force Development
SPJ	Spike Jump
SSC	Stretch-shortening cycle
SWC	Smallest Worthwhile Change
WT	Weight Training
