# Supplementary material for: Vertical Jump Changes Across the Stretch-Shortening-Cycle Continuum Following Complex Training in Adolescent Female Volleyball Players: A Single-Arm Bayesian Analysis
Source: Sports (Basel). 2026 Jun 26;14(7):267. doi: 10.3390/sports14070267 (PMC13417267; doi:10.3390/sports14070267)
Supplement: Supplementary file 1 [file sports-14-00267-s001.zip › sports-4383206-supplementary.pdf]

Supplementary Materials

Vertical Jump Changes Across the Stretch-Shortening-Cycle Continuum Following Complex Training in Adolescent Female Volleyball Players: A Single-Arm Bayesian Analysis

Table S1. Prior-sensitivity analysis. Bayes Factors (BF_{10}) for each outcome computed with the Bayesian paired-samples (JZS) t-test across four Cauchy prior scales (r). The default analysis reported in the main text uses r = 0.707.

Outcome	r = 0.5	r = 0.707	r = 1.0	r = 1.414
Block Volleyball Jump height	24.2	29.7	34.3	36.8
Countermovement Jump height	22.3	27.2	31.3	33.3
Spike Jump height	97.4	126.9	157.9	184.1
Body mass	0.43	0.34	0.26	0.19
Muscle mass (%)	1.62	1.56	1.41	1.19
Fat mass (%)	1.17	1.07	0.93	0.76

Note. $BF_{10} > 1$ indicates evidence for a change; $BF_{10} < 1$ indicates evidence for no change (for example, $BF_{10} = 0.34$ corresponds to $BF_{01} \approx 3.0$). Conventional bands (Jeffreys): 1–3 anecdotal, 3–10 moderate, 10–30 strong, 30–100 very strong, > 100 decisive. Across all four priors the evidence for a change in each jump height remained at least strong ($BF_{10} > 10$), and the three body-composition outcomes remained anecdotal (BF_{10} close to 1). As the prior scale widens, the precise band for the jumps shifts upward (for example, the Spike Jump moves from very strong to decisive), as expected when more prior mass is placed on larger effects, but the qualitative conclusions are unchanged. Analyses were performed in R (BayesFactor package) and independently reproduced in Python (PyMC).
